# Supplementary material for: Identification of Novel Susceptibility Genes for Early-Onset Colorectal Cancer Through Germline Rare Variant Burden Testing
Source: Cancers (Basel). 2025 Dec 9;17(24):3931. doi: 10.3390/cancers17243931 (PMC12731108; doi:10.3390/cancers17243931)
Supplement: Supplementary file 1 [file cancers-17-03931-s001.zip › cancers-4013320-supplementary.pdf]

# Supplementary Materials: Identification of Novel Susceptibility Genes for Early-Onset Colorectal Cancer through Germline Rare Variant Burden Testing

**Ruocen Song, Reger R. Mikaeel, Zhongping He, Mehgan Horsnell, Wendy Uylaki, Weimin Meng, Nicola K. Poplawski, Bernd Wollnik, Yun Li, Jinghua Feng, Hamish S. Scott, Yufeng Shen, Chen Wang, Rui Yin, Yousong Ding, Xavier Lior, Wendy K. Chung, Eric Smith, Timothy J. Price, Joanne P. Young, and Xiao Fan**

(A)

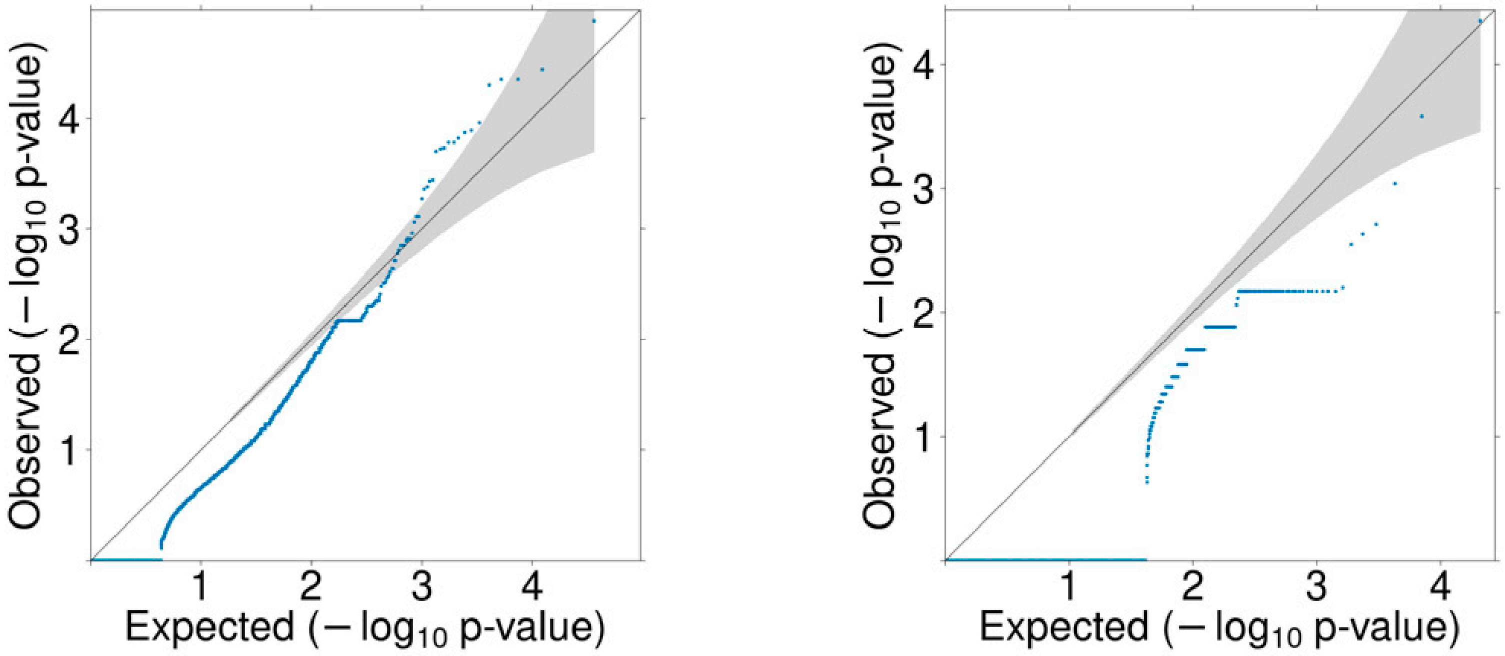


(B)

| Variant Type | # of Variants per Individual in SAYO | # of Variants per Individual in SPARK | Relative Risk | *P* Value |
|---|---|---|---|---|
| Synonymous | 26.43 | 26.65 | 0.99 | 0.27 |
| Inframe indels | 1.23 | 1.14 | 1.07 | 0.30 |

**Figure S1.** Post quality control for rare synonymous variants and inframe indels in SAYO and SPARK. (**A**) QQ plot for rare synonymous and inframe indel variants after quality control. Each blue dot represents a gene, and the 95% confidence intervals are shaded. Left: synonymous variants; Right: inframe indels. (**B**) Non-functional variant burden comparison between SAYO and SPARK. # refers number.

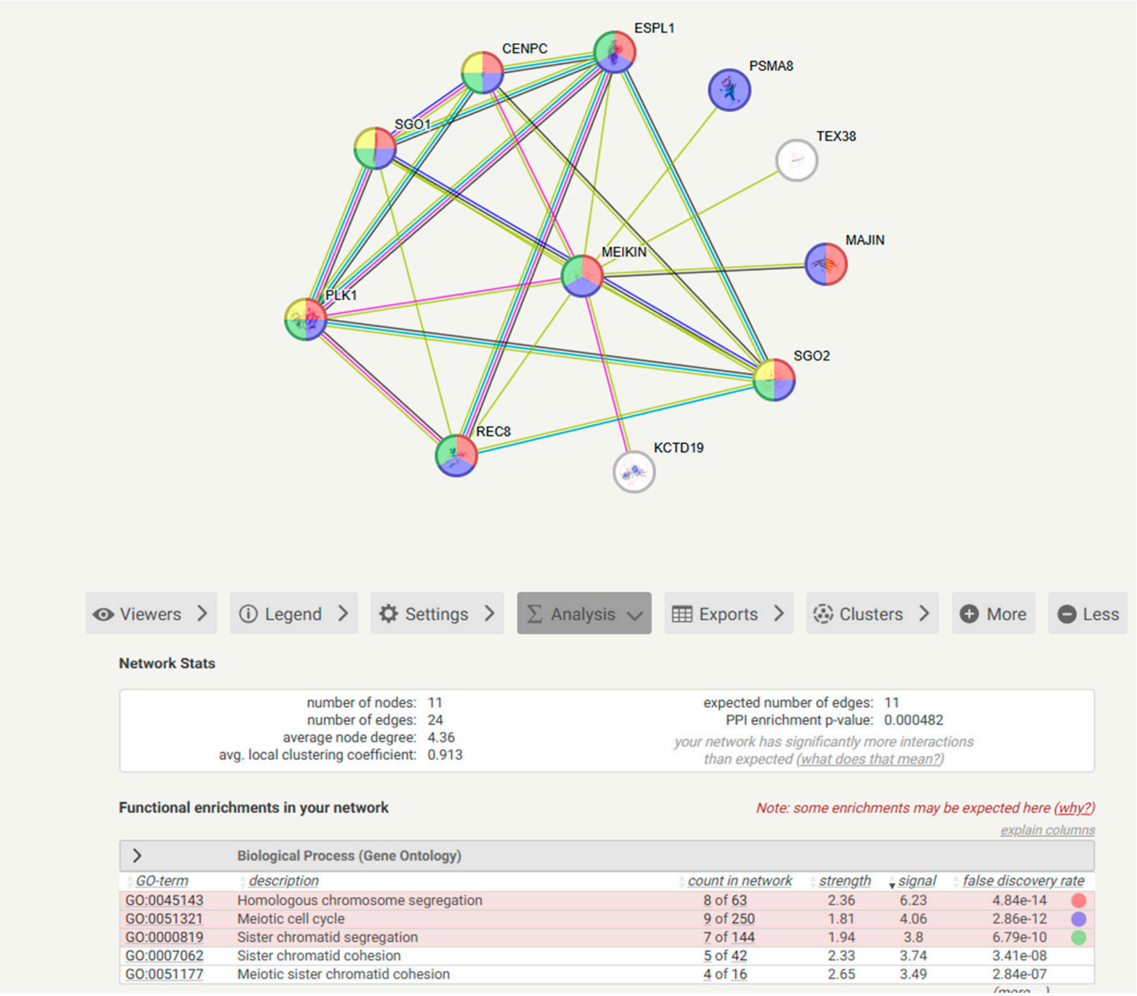


**Figure S2.** Protein–Protein Interaction Network of *MEIKIN*-Associated Genes. Image accessed on May 19 2025: https://string-db.org/cgi/network?taskId=bVRYnqmbo9OL&sessionId=zlSRVlopTeaz

**Table S1.** Quality control thresholds for variant filtering in SAYO and SPARK cohorts.

| | SAYO | SPARK | UKBB |
|---|---|---|---|
| **Maximum allele frequency in gnomAD** | <0.0001 | <0.0001 | <0.0001 |
| **allele frequency in the UK Biobank** | <0.0001 | <0.0001 | <0.0001 |
| **Genotype Quality** | >10 | >10 | >25 |
| **Allele depth for alternative alleles** | >2 | >2 | >3 |
| **Total read depth** | >7 | >7 | >10 |
| **Allele Balance** | >=0.1 | >=0.1 | >=0.2 |
| **Quality Filter** | VQSLOD > 2 | QUAL > 38 | QUAL > 30 |

Abbreviations: gnomAD: genome aggregation database; VQSLOD: Variant Quality Score Log-Odds; QUAL: Phred-scaled Quality Score.

**Table S2.** 21 colorectal cancer risk genes based on the ASCO guidelines.

| | | | | | | |
|---|---|---|---|---|---|---|
| *EPCAM* | *SMAD4* | *STK11* | *MBD4* | ***GREM1*** | *MSH3* | *PTEN* |
| *RNF43* | *APC* | *AXIN2* | *PMS2* | *MLH1* | *POLD1* | *CHEK2* |
| *POLE* | *NTHL1* | *TP53* | *MSH6* | *BMPR1A* | *MUTYH* | *MSH2* |

Oncogenes are in bold font.

**Table S3.** 42 colorectal cancer risk genes based on the OMIM guidelines.

| | | | | | | |
|---|---|---|---|---|---|---|
| ***PLA2G2A*** | *MUTYH* | ***NRAS*** | ***CRCS5*** | *BMPR1A* | ***CCND1*** | ***CRCS7*** |
| *POLE* | ***CRCS8*** | *MLH3* | ***AKT1*** | ***GREM1*** | ***BUB1B*** | *NTHL1* |
| ***CRCS9*** | *TP53* | *FLCN* | *RNF43* | *AXIN2* | ***SMAD7*** | *SMAD4* |
| *DCC* | *BAX* | *POLD1* | ***BUB1*** | ***CRCS11*** | *CHEK2* | *EP300* |
| *TGFBR2* | ***CTNNB1*** | ***PIK3CA*** | ***FGFR3*** | ***TLR2*** | *MSH3* | *APC* |
| *MCC* | ***BRAF*** | *DLC1* | *PDGFRL* | ***CRCS6*** | ***CRCS2*** | ***GALNT12*** |

Oncogenes are in bold font.

**Table S4.** Individual variants information for known CRC risk genes.

| Gene | Sample ID | Variant (chr:pos ref>alt) | Type | VEST4 | QUAL | AD1 | AD2 | Gender | Age at Diagnosis | Case Status | Sub Phenotype |
|---|---|---|---|---|---|---|---|---|---|---|---|
| *AKT1* | SAYO019197 | chr14:104773915 GC>G | frameshift | 1.00 | 5.43 | 10 | 3 | M | 42 | CRC | Rectal |
| *APC* | SAYO019192 | chr5:112754922 A>T | missense | 0.71 | 21.26 | 30 | 21 | M | 42 | CRC | Rectal |

| | | | | | | | | | | | |
|---|---|---|---|---|---|---|---|---|---|---|---|
| *AXIN2* | SAYO020243 | chr17:65558148 C>G | missense | 0.76 | 20.09 | 113 | 86 | M | | SP | Serrated polyposis syndrome |
| *BRAF* | SAYO020246_A | chr7:140794414 G>C | missense | 0.64 | 21.50 | 59 | 33 | | | CRC | Caecal |
| *CHEK2* | SAYO020223 | chr22:28719471 C>CA | frameshift | 1.00 | 6.81 | 25 | 33 | F | | SP | Serrated polyposis syndrome |
| *DCC* | SAYO018059 | chr18:52340852 T>C | missense | 0.51 | 20.81 | 44 | 25 | M | 34 | CRC | Colon cancer |
| | SAYO021289 | chr18:53467938 A>T | missense | 0.83 | 21.03 | 35 | 38 | M | | SP | Serrated polyposis syndrome |
| *DLC1* | SAYO016035 | chr8:13100734 T>C | missense | 0.92 | 21.64 | 31 | 23 | F | 42 | CRC | Transverse colon |
| | SAYO015017 | chr8:13100050 G>C | missense | 0.91 | 21.83 | 36 | 55 | F | 52 | CRC | Ascending colon |
| | SAYO020244 | chr8:13099571 C>G | missense | 0.55 | 21.69 | 51 | 56 | F | | SP | Sessile serrated adenomas |
| *FGFR3* | SAYO020214 | chr4:1805561 G>A | missense | 0.74 | 21.18 | 34 | 15 | F | | SP | Serrated polyposis syndrome |
| *MCC* | SAYO016042 | chr5:113122778 T>A | missense | 0.76 | 21.02 | 29 | 50 | F | 48 | CRC | Transverse colon |
| | SAYO019102 | chr5:113064033 C>T | missense | 0.59 | 21.68 | 98 | 98 | M | 34 | CRC | Colon cancer |
| | SAYO019128 | chr5:113085169 C>T | missense | 0.85 | 21.13 | 58 | 48 | M | 30 | SP | Serrated polyposis syndrome |
| *MLH1* | SAYO019176 | chr3:37028927 A>AT | frameshift | 1.00 | 6.33 | 19 | 15 | F | 16 | CRC | Lynch syndrom |
| *MLH3* | SAYO019139 | chr14:75049580 C>A | missense | 0.85 | 20.41 | 110 | 76 | M | 36 | CRC | NA |
| | SAYO019193 | chr14:75049650 G>C | missense | 0.81 | 21.75 | 27 | 28 | M | 32 | CRC | Rectal |
| *MSH2* | SAYO020237 | chr2:47414368 C>T | stop_gained | 1.00 | 21.99 | 26 | 27 | F | 59 | CRC | NA |

| | | | | | | | | | | | |
|---|---|---|---|---|---|---|---|---|---|---|---|
| | SAYO021271 | chr2:47475254 G>A | missense | 0.66 | 21.14 | 27 | 19 | M | 28 | CRC | NA |
| | SAYO020218 | chr2:47429919 A>G | missense | 0.56 | 21.33 | 24 | 34 | F | 26 | CRC | NA |
| | SAYO019160 | chr2:47403196 C>T | missense | 0.54 | 21.25 | 53 | 29 | F | 44 | CRC | Rectal |
| | SAYO019167 | chr2:47403211 A>G | missense | 0.51 | 21.24 | 60 | 60 | M | 45 | CRC | Rectal |
| *MSH6* | SAYO019159 | chr2:47800909 C>T | missense | 0.94 | 19.52 | 48 | 41 | F | 53 | CRC | Colon cancer |
| | SAYO021280 | chr2:47800965 C>G | stop_gained | 1.00 | 22.09 | 37 | 30 | M | 32 | CRC | Rectal |
| | SAYO021253 | chr2:47799403 G>C | missense | 0.83 | 21.84 | 138 | 131 | F | 19 | CRC | Colon cancer |
| | SAYO019150 | chr2:47803552 CT>C | frameshift | 1.00 | 6.42 | 47 | 43 | M | 54 | CRC | NA |
| *MUTYH* | SAYO018079 | chr1:45332080 G>A | stop_gained | 1.00 | 20.45 | 110 | 107 | F | 29 | CRC | NA |
| | SAYO016042 | chr1:45331676 G>A | stop_gained | 1.00 | 21.36 | 154 | 121 | F | 48 | CRC | Transverse colon |
| | SAYO019136 | chr1:45331237 C>T | missense | 0.74 | 21.66 | 76 | 70 | M | 46 | CRC | Rectum |
| *NTHL1* | SAYO018098 | chr16:2044738 C>T | missense | 1.00 | 21.48 | 134 | 111 | F | 44 | CRC | NA |
| *PIK3CA* | SAYO019116 | chr3:179199701 G>A | missense | 0.96 | 20.87 | 42 | 32 | M | 44 | CRC | Rectal |
| *POLE* | SAYO021283 | chr12:132639209 C>G | missense | 1.00 | 21.14 | 63 | 46 | M | 36 | CRC | Rectal |
| | SAYO019136 | chr12:132659430 C>G | missense | 0.77 | 20.59 | 77 | 70 | M | 46 | CRC | Rectal |
| | SAYO015002 | chr12:132636005 AG>A | frameshift | 1.00 | 6.36 | 37 | 42 | F | 39 | CRC | Sigmoid colon |
| | SAYO019192 | chr12:132677599 GGA>G | frameshift | 1.00 | 4.56 | 24 | 20 | M | 42 | CRC | Rectal |
| | SAYO021287 | chr12:132668888 G>A | missense | 0.93 | 21.79 | 29 | 29 | F | | SP | Sessile serrated adenoma |

Abbreviations: CRC: colorectal cancer; SP: significant polyps; CHR: chromosome; POS: position; REF/ALT: reference and alternate alleles; TYPE: variant type; VEST4: predicted pathogenicity score; QUAL: variant quality score; AD1/AD2: read depth for REF and ALT alleles; F: female; M: male. .

**Table S5.** Individual variants information for novel CRC risk-susceptible genes.

| Gene | Sample ID | Variant (chr:pos ref>alt) | Type | VEST 4 | QUAL | AD1 | AD2 | Case Status | Sub Pheno-typ | Co-occurrence w/ CRC Risk Genes | Gen-der | Family History of CRC or Polyps | Family History of Other Types of Cancer | MMR IHC Status | Age at Diag-nosis |
|---|---|---|---|---|---|---|---|---|---|---|---|---|---|---|---|
| *MEI-KIN* | SAYO015002 | chr5:131911860 G>C | missense | 0.24 | 21.09 | 39 | 31 | CRC | *Sig-moid colon* | *POLE* | F | Yes Polyps | Yes | Nor-mal | 39 |
| | SAYO019188 | chr5:131944742 C>G | missense | 0.11 | 21.72 | 20 | 19 | CRC | NA | | M | No | No | Nor-mal | 51 |
| | SAYO021284 | chr5:131944710 AT>A | frameshift | 1.00 | 4.02 | 31 | 17 | SP | Sessile ser-rated adeno-mas | | F | CRC and polyps | Yes | NA | 28 |
| | SAYO021290 | chr5:131921915 GTTCT>G | frameshift | 1.00 | 6.6 | 32 | 29 | SP | Ser-rated poly-posis syn-drome | | F | CRC | Yes | NA | 24 |
| *STK25* | SAYO019122 | chr2:241498687 C>T | stop_gained | 1.00 | 20.53 | 105 | 89 | CRC | Colon cancer | | F | Yes Polyps | Yes | NA | 46 |
| | SAYO019193 | chr2:241498724 A>AG | frameshift | 1.00 | 6.26 | 24 | 29 | CRC | *Rectal* | *MLH3* | M | CRC and Polyps | Yes | NA | 32 |
| | SAYO020206 | chr2:241499173 del | splice | 1.00 | 5.38 | 86 | 31 | CRC | NA | | F | No | Yes | Nor-mal | 38 |
| *PGBD4* | SAYO020220 | chr15:34104284 T>A | missense | 0.38 | 21.86 | 25 | 35 | CRC | NA | | M | No | No | Nor-mal | 54 |
| | SAYO020214 | chr15:34103574 C>T | missense | 0.31 | 21.74 | 24 | 32 | SP | Ser-rated poly-posis syn-drome | *FGFR3* | F | No | Yes | NA | 31 |

| | | | | | | | | | | | | | | | |
|---|---|---|---|---|---|---|---|---|---|---|---|---|---|---|---|
| | SAYO019128 | chr15:34104237 C>T | missense | 0.28 | 21.04 | 85 | 76 | SP | Serrated polyposis syndrome | *MCC* | M | Yes Polyps | Yes | NA | 30 |
| | SAYO021253 | chr15:34103027 G>A | missense | 0.23 | 20.18 | 125 | 117 | CRC | *NA* | *MSH6* | F | Yes CRC | Yes | PMS-2 & MLH-1 absent | 53 |
| *DIRAS3* | SAYO020207 | chr1:68046735 A>T | missense | 0.94 | 21.6 | 42 | 34 | CRC | Colon cancer | | M | Yes CRC | Yes | MLH-1 absent | 51 |
| | SAYO019125 | chr1:68046780 C>T | stop_gained | 1.00 | 21.71 | 17 | 21 | CRC | Colon cancer | | F | No | No | Normal | 50 |
| | SAYO020222 | chr1:68047187 ATC>A | frameshift | 1.00 | 6.71 | 40 | 50 | CRC | Sigmoid | | F | Yes CRC | Yes Polyps | Normal | 52 |
| *ATG3* | SAYO018076 | chr3:112561480 C>T | missense | 0.78 | 21.23 | 15 | 5 | SP | Sessile serrated adenomas | | M | CRC and polyps | Yes | Normal | 32 |
| | SAYO019106 | chr3:112536529 A>G | missense | 0.66 | 21.54 | 31 | 27 | CRC | NA | | M | Yes Polyps | Yes | Normal | 38 |
| | SAYO015026 | chr3:112538158 C>G | missense | 0.53 | 21.41 | 24 | 18 | SP | Sessile serrated lesion | | M | Yes Polyps | Yes | NA | 42 |
| | SAYO019152 | chr3:112541841 T>A | missense | 0.51 | 21.21 | 45 | 22 | CRC | Rectal | | F | No | Yes | Normal | 30 |
| *RPS6KA4* | SAYO021283 | chr11:64360289 A>C | missense | 0.96 | 20.24 | 26 | 13 | CRC | *Rectal* | *POLE* | M | Yes CRC | Yes | Normal | 36 |
| | SAYO018085 | chr11:64370626 T>A | missense | 0.86 | 21.64 | 60 | 46 | CRC | NA | | M | Yes CRC | Yes | Normal | 45 |
| | SAYO019180 | chr11:64368205 CT>C | frameshift | 1.00 | 4.54 | 13 | 3 | CRC | NA | | F | Yes CRC | Yes | Normal | 52 |
| *DDX42* | SAYO019167 | chr17:63808938 T>C | missense | 0.90 | 21.18 | 27 | 18 | CRC | NA | | M | No | Yes | NA | 45 |

| | | | | | | | | | | | | | |
|---|---|---|---|---|---|---|---|---|---|---|---|---|---|
| SAYO0191 16 | chr17:63808863 G>A | missense | 0.88 | 21.55 | 18 | 20 | CRC | NA | M | Yes | Yes | NA | 44 |
| SAYO0191 92 | chr17:63811988 G>A | stop_gained | 1.00 | 21.44 | 29 | 21 | CRC | NA | M | Yes Polyps | Yes | NA | 42 |

Abbreviations: CRC: colorectal cancer; SP: significant polyps; CHR: chromosome; POS: position; REF/ALT: reference and alternate alleles; TYPE: variant type; VEST4: predicted pathogenicity score; QUAL: variant quality score; AD1/AD2: read depth for REF and ALT alleles; F: female; M: male.

**Table S6.** Burden test results for *MEIKIN* in the UKBB WGS cohort using a fixed VEST4 threshold across cancer types.

| Cancer Type | # of Variants per Individual in Cases | # of Variants per Individual in Controls | Relative Risk | VEST4 Threshold | *P* Value | Analysis |
|---|---|---|---|---|---|---|
| **Proximal group** | 0.0004 | 0.0014 | 0.245 | 0.109 | 0.983 | Missense |
| | 0.0004 | 0.0017 | 0.204 | 0.109 | 0.993 | LoF and missense |
| | 0.0000 | 0.0002 | 0.000 | N/A | 1.000 | LoF |
| **Distal group** | 0.0016 | 0.0014 | 1.095 | 0.109 | 0.440 | Missense |
| | 0.0023 | 0.0017 | 1.318 | 0.109 | 0.198 | LoF and missense |
| | 0.0007 | 0.0003 | 2.458 | N/A | 0.086 | LoF |

Abbreviations: proximal group: proximal colon cancer; distal group: distal colon cancer, rectosigmoid junction cancer, rectal cancer. # refers number.

**Table S7.** Burden test results for *MEIKIN* in the UKBB WGS cohort using variable VEST4 thresholds across cancer types.

| Cancer Type | # of Variants per Individual in Cases | # of Variants per Individual in Controls | Relative Risk | VEST4 Threshold | *P* Value | Analysis |
|---|---|---|---|---|---|---|
| **Proximal group** | 0.0000 | 0.0000 | N/A | 0.874 | 1.000 | Missense |
| | 0.0000 | 0.0000 | N/A | 1.001 | 1.000 | LoF and missense |
| | 0.0000 | 0.0000 | N/A | N/A | 1.000 | LoF |
| **Distal group** | 0.0016 | 0.0009 | 1.830 | 0.240 | 0.222 | Missense |
| | 0.0023 | 0.0011 | 1.982 | 0.240 | **0.048** | LoF and missense |
| | 0.0007 | 0.0003 | 2.458 | N/A | 0.096 | LoF |

Abbreviations: proximal group: proximal colon cancer. distal group: distal colon cancer, rectosigmoid junction cancer, rectal cancer. # refers number.
